# Supplementary material for: Preclinical safety evaluation of continuous UV-A lighting in an operative setting
Source: PLoS One. 2023 Nov 17;18(11):e0291083. doi: 10.1371/journal.pone.0291083 (PMC10656011; doi:10.1371/journal.pone.0291083)
Supplement: S1 Fig — (A) Raw blot images from Fig 3A with ladders shown, the rightmost lane was the negative control, untreated HEK293 cells and was not used in the final figure for publication. (B) Raw blot images from Fig 3B with ladders shown, the rightmost lane was the negative control, controls of HEK293 exposed to UV in a biosafety hood for 2 hours and untreated HEK293 cells were not used in the final figure for publication. (PDF) [file pone.0291083.s001.pdf]

A

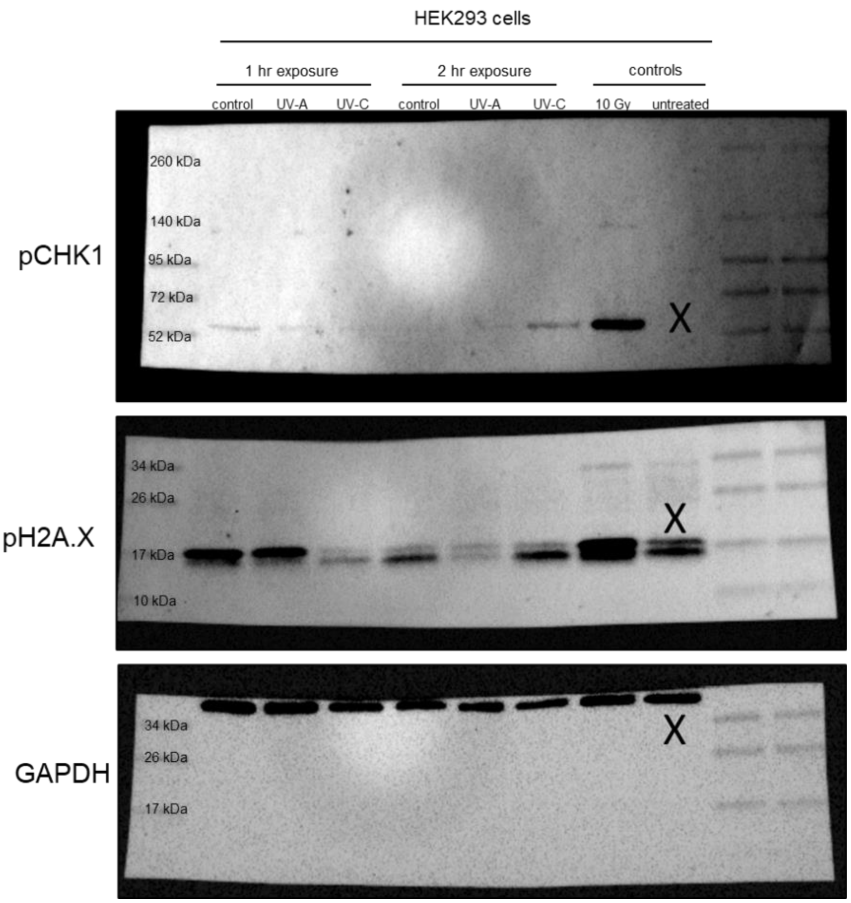

B

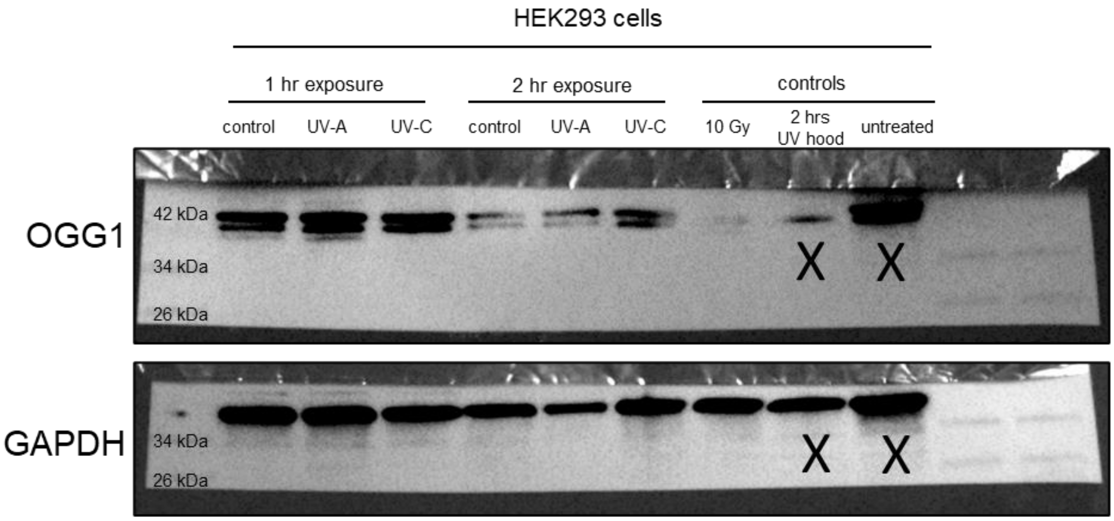

Figure S1. Raw western blot images from Figure 1. (A) Raw blot images from Figure 1A with ladders shown, the rightmost lane was the negative control, untreated HEK293 cells and was not used in the final figure for publication. (B) Raw blot images from Figure 1B with ladders shown, the rightmost lane was the negative control, controls of HEK293 exposed to UV in a biosafety hood for 2 hours and untreated HEK293 cells were not used in the final figure for publication.
