# Supplementary material for: Preclinical safety evaluation of continuous UV-A lighting in an operative setting
Source: PLoS One. 2023 Nov 17;18(11):e0291083. doi: 10.1371/journal.pone.0291083 (PMC10656011; doi:10.1371/journal.pone.0291083)
Supplement: S1 Table — (DOCX) [file pone.0291083.s002.docx]

| **Measured PPE** | **Brand** | **Reference ID** |
| --- | --- | --- |
| Drape leggings | Medline Clear Leggings 33” x 49” with 6” cuff (15 cm) | DYNJP2462 |
| Eyeglasses | TIDI Shield (Grab ‘n Go ® Eye Shields) | 9210A-100 |
| Surgeons Cap | CardinalHealth Surgeon’s Cap | 4359 |
| Bouffant Cap | CardinalHealth Comfort Bouffant Large 24 inches | 3274 |
| Light Blue Gown | Medline Prevention Plus Surgical Gown XL | DYNJP2302P |
| Dark Blue Gown | Medline SIRUS Surgical Gown XL | DYNJP2402 |
| Surgical Mask (blue) | CardinalHealth Surgical Mask with Anti-Fog Foam | AT71235 |
| Surgical Mask (green) | CardinalHealth Surgical Mask with Anti-Fog Film | AT752005 |
| Drape Sheet | Medline Ultragard | DYNJP1050UG |
| Polyisoprene Surgical Glove | CardinalHealth Protexis Size 8 | 2D72PT80X |

*Table S1. Brand information and Reference IDs for PPE used in the UV-A transmission, transmittance, and absorbance study.*
